# Regulation of Plant Immunity by Nuclear Membrane-Associated Mechanisms

**DOI:** 10.3389/fimmu.2021.771065

**Published:** 2021-12-06

**Authors:** Yiling Fang, Yangnan Gu

**Affiliations:** ^1^ Department of Plant and Microbial Biology, University of California, Berkeley, CA, United States; ^2^ Innovative Genomics Institute, University of California, Berkeley, CA, United States

**Keywords:** nuclear envelope (NE), nuclear pore complex (NPC), nuclear transport receptors (NTRs), nucleoskeletal proteins, innate immune system, plant Immunity, nucleocytoplasmic continuum, nuclear lamina

## Abstract

Unlike animals, plants do not have specialized immune cells and lack an adaptive immune system. Instead, plant cells rely on their unique innate immune system to defend against pathogens and coordinate beneficial interactions with commensal and symbiotic microbes. One of the major convergent points for plant immune signaling is the nucleus, where transcriptome reprogramming is initiated to orchestrate defense responses. Mechanisms that regulate selective transport of nuclear signaling cargo and chromatin activity at the nuclear boundary play a pivotal role in immune activation. This review summarizes the current knowledge of how nuclear membrane-associated core protein and protein complexes, including the nuclear pore complex, nuclear transport receptors, and the nucleoskeleton participate in plant innate immune activation and pathogen resistance. We also discuss the role of their functional counterparts in regulating innate immunity in animals and highlight potential common mechanisms that contribute to nuclear membrane-centered immune regulation in higher eukaryotes.

## Introduction

The innate immune system in plants and animals arose independently but converged with a similar set of molecular tools for pathogen perception (1, 2). Higher plants possess an enormous number of surface-localized as well as intracellular-distributed immune receptors that recognize a variety of immunological signals associated with pathogen infections. Surface-localized plant immune receptors consist mostly of plasma membrane-anchored receptor kinases and receptor-like proteins that are conceptually analogous to Toll-like receptors in animals. They are referred to as pattern recognition receptors (PRRs) and are able to detect specific microbe-associated molecular patterns (MAMPs, e.g. bacterial flagellin and lipopolysaccharides) or host-derived, damage-associated molecular patterns (DAMPs, e.g. cutin and apoplastic peptide fragments) through their extracellular leucine-rich repeat domains (3, 4). Once activated, PRRs engage diverse signaling cascades including mitogen-activated protein kinase (MAPK)-, Ca^2+^-, and reactive oxygen species (ROS)-mediated signaling to activate pattern-triggered immunity (PTI), a prominent and first layer of active immune response in plants (5–7). However, some pathogens evolved functionally versatile effector proteins, which are delivered into plant cells to target critical immune regulators and compromise PTI signaling, leading to effector-triggered susceptibility (ETS) (8). For example, the effector protein AvrPto from bacterial pathogen *Pseudomonas syringae* binds PRRs (e.g. FLAGELLIN SENSITIVE 2) and their signaling partner (e.g. BRASSINOSTEROID INSENSITIVE 1-ASSOCIATED KINASE 1) to block PTI signaling (9, 10). To counteract ETS, a group of intracellular immune receptors that belong to the nucleotide-binding, leucine-rich repeat (NLR) superfamily evolved to activate the second layer of plant immunity. Pathogen effectors can be directly or indirectly recognized by cognate NLRs, which activate a strong and robust immune response termed effector-triggered immunity (ETI) (11, 12). Activated plant NLRs are assembled into resistosomes (13, 14), which form permeable calcium channels on the plasma membrane to activate defense (15) or mediate NAD+ cleavage to promote cell death (16–18).

PTI and ETI appear to have distinct early signaling but share many common downstream immune regulators to coordinate transcriptional reprogramming toward defense activation. Recent advances also revealed that PTI and ETI are inter-dependent and mutually enhanced to achieve full resistance against pathogens (19–21). Immune activation in local infected cells or tissues can further induce systemic acquired resistance (SAR), an immune mechanism that primes neighboring and distal cells and tissues for an enhanced and broad-spectrum resistance against future pathogen infection. Induction of SAR relies on phytohormone signaling including salicylic acid (SA) and jasmonic acid (JA) (11, 22). Activation of PTI, ETI, and SAR all requires efficient and highly regulated nucleocytoplasmic exchange of signals. As the major communication interface between the cytoplasm and the nucleus, the nuclear envelope (NE) evolved as a critical platform to integrate, decode, transmit, and respond to immune signals. In this review, we focus on discussing the functions of NE-associated proteins in regulating plant innate immunity and comparing their roles in NE-based immune regulatory mechanisms between plants and animals.

## Extensive Involvements of Nucleoporins in Plant Immune Regulation

**Figure 1:**
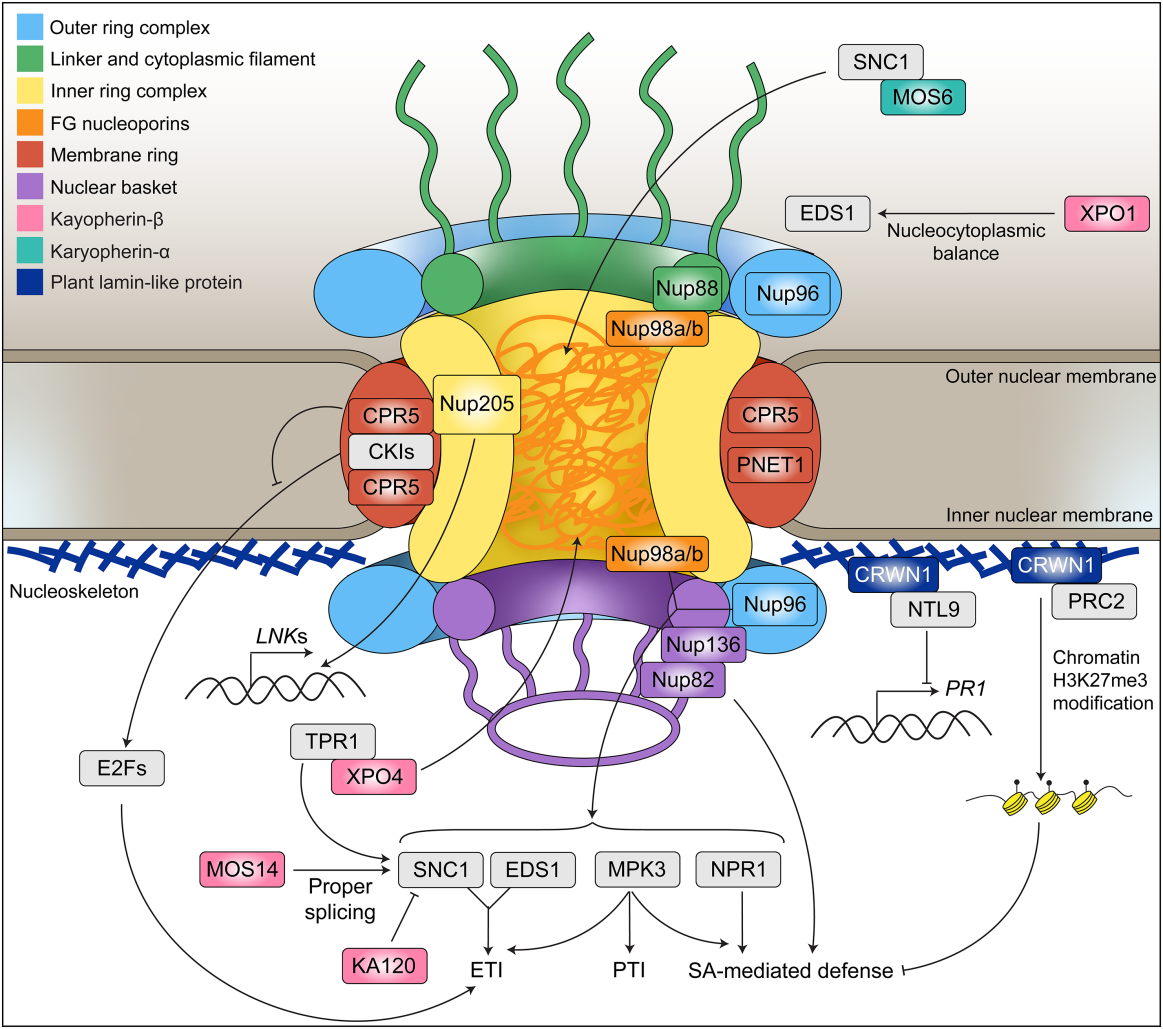
Summary of the regulatory roles of nuclear membrane-associated proteins in plant immune pathways. Critical plant immune regulators that are subject to regulation by nuclear membrane-associated mechanisms include the NLR-type immune receptor SNC1 that activates effector-triggered immunity (ETI), the critical ETI signaling component EDS1 that acts downstream of SNC1, transcription corepressor TPL and TPRs that work together with SNC1 to activate defense gene expression, cell cycle transcription factor E2Fs that activate ETI in a noncanonical manner, the master regulator of SA-mediated immunity NPR1, the mitogen-activated protein kinase MPK3 that widely participates in various types of plant immune responses including PTI, ETI, and basal resistance, core clock regulator LNKs that are required for basal resistance, and the transcription factor NTL9 that represses defense gene (e.g. *PR1*) expression. The outer ring complex (ORC) of the nuclear pore complex (NPC) is required for SNC1-mediated immune activation and basal resistance. As components of the central channel barrier, FG nucleoporin Nup98 and Nup88 are required for nuclear accumulation of SNC1, EDS1, NPR1, and MPK3. The membrane nucleoporin CPR5 (part of the NPC membrane ring) gates ETI activation by sequestering cyclin kinase inhibitor SIM/SMR1 and preventing E2F activation. Nup205 is required for the proper expression of *LNK* family genes and activation of basal resistance. Nup82 and Nup136 are involved in SA-mediated defense activation. Karyopherin-α MOS6 serves as the possible importin adapter for multiple NLR proteins, including SNC1 and TN13. Karyopherin-β KA120 is required for suppressing the nuclear activity of SNC1 and prevents SNC1 autoimmune activation. XPO4 mediates TPR1 nuclear export and negatively regulates SA-mediated immune amplification during ETI. MOS14 is required for proper splicing of NLR genes, including SNC1. The nucleoskeleton protein CRWN1 plays a role in suppressing both SA responses through interacting with transcription factor NTL9 that represses defense gene *PR1* expression and interacting with epigenetic regulator PRC2 that facilitates the H3K27me3 of genes promoting SA synthesis.

Nuclear pore complexes (NPC) perforate the double-layered NE and provide physical access for molecules to travel in or out of the nucleus. Each NPC is a mega protein complex and is assembled by 500~1,000 nucleoporin proteins (Nups) of ~40 different kinds (23–25). These nucleoporins build different NPC modules, including the inner ring complex (IRC) and the outer ring complex (ORC) that together form an octagonal symmetric core scaffold, the membrane ring composed by transmembrane nucleoporins that anchors the core scaffold to the nuclear pore membrane, the central channel barrier composed by phenylalanine-glycine (FG)-rich nucleoporins that fills the core scaffold and mediate selective cargo transport, and the nuclear basket and cytoplasmic filaments that extrude from the core scaffold and establish connections with the nucleoplasmic and cytoplasmic contents, respectively (26–28) (**Figure 1**). Extensive evidence supports that NPC integrity is essential for activating innate immune responses in both plants and animals. Interestingly though, different nucleoporins appear to play distinct roles in immune regulation and show functional specificity.

The Arabidopsis *SUPPRESSOR OF NPR1-1, CONSTITUTIVE 1 (SNC1)* gene encodes an NLR protein that undergoes nuclear translocation for immune signaling, and enhanced nuclear accumulation of SNC1 leads to autoimmune activation (29, 30). Elegant genetic studies showed that several conserved ORC scaffolding nucleoporins, including MOS3 (MODIFIER OF SNC1 3)/Nup96, Nup160, and Seh1, are required for SNC1-mediated autoimmune induction as well as basal resistance against *P. syringae* (31, 32) (**Figure 1**). In animals, Nup96 was shown to be important for both innate and adaptive immune pathways. Defects in Nup96 were reported to impair interferon-γ-mediated induction of major histocompatibility complexes (MHC I and MHC II), which are essential for antigen presentation. Mutations in *Nup96* also diminished MHC-dependent T cell proliferation in mice (33). Although molecular mechanisms behind Nup96-dependent immune regulation remain largely obscure in both animals and plants, the ORC is essential for the proper assembly of the NPC core scaffold and bulk mRNA export (34–36). It is conceivable that loss of ORC nucleoporins may lead to major structural and functional defects in the NPC, which compromise nucleocytoplasmic exchange of critical immune signals and the newly transcribed immune-related mRNA population.

The FG nucleoporins that contain intrinsically disordered FG-rich regions distribute along the central channel of the NPC and form the molecular barrier to enable selective cargo transport. At least six FG nucleoporins have been reported to play a role in regulating the innate immune response in Arabidopsis (37). Among them, Nup98, a conserved FG nucleoporin, has been characterized in detail. Arabidopsis encodes two Nup98 paralogs, Nup98a and Nup98b, both of which interact with a conserved non-FG nucleoporin Nup88/MOS7. A partial loss of function mutation in Arabidopsis *Nup88* (*mos7-1*) results in compromised resistance against both bacterial and fungal pathogens. The *mos7-1* mutation specifically attenuates the nuclear accumulation of a series of important plant immune regulators, including a key ETI signaling protein ENHANCED DISEASE SUSCEPTIBILITY 1 (EDS1), the master regulator of SA-mediated defense response NONEXPRESSOR OF PATHOGENESIS-RELATED GENES 1 (NPR1), MITOGEN-ACTIVATED PROTEIN KINASE 3 (MPK3), and SNC1, but not other nuclear proteins such as histone protein and transcription factor CELL DIVISION CYCLE 5 (CDC5) and TGA2 (38, 39) (**Figure 1**). It was hypothesized that the *mos7-1* mutation disrupts the Nup98-Nup88 interaction, which alters the NPC permeability and leads to disruption of the nuclear transport of immune-related cargoes. Consistent with this hypothesis, both *nup98a* and *nup98b* mutants are more susceptible to the fungal pathogen *Botrytis cinerea* than WT plants (39), suggesting a specialized function of Nup98 in plant immunity. In line with the observation in Arabidopsis, a rice Nup98 homolog, APIP12, was shown to be important for basal resistance against the rice blast fungus *Magnaporthe oryzae*. Notably, an *M. oryzae* effector AvrPiz-t evolved to specifically target APIP12 to enhance pathogen virulence (40), highlighting its functional significance in basal defense activation. Knocking out *APIP12* results in an increase in lesion density associated with *M. oryzae* infection. In Drosophila, Nup98 was reported to play a role in defense against RNA viral infection. However, rather than functioning at the NPC, *Dm*Nup98 is re-located from the NPC to the nucleoplasm upon viral infection and directly binds promoters of antiviral genes to facilitate their expression (41).

Both Nup98 and Nup96 discussed above are functionally conserved nucleoporins and are encoded by the same gene and post-translationally processed into two proteins in animals and fungi (42, 43). However, in plants, except *Chlamydomonas reinhardtii* and *Physcomitrella patens*, Nup98 and Nup96 are encoded by separate genes, which are, more often than not, located on different chromosomes. Arabidopsis encodes one Nup96 and two Nup98 proteins as mentioned above, and *Oryza sativa* encodes one Nup96 and three AtNup98a-like proteins, including APIP12. Duplication of *Nup98* likely occurred from mosses as *Physcomitrella patens* contains two copies of *Nup98*, one encoded with *Nup96* and the other located on a separate chromosome. Therefore, it appears that *Nup98* not only separated from *Nup96* but also gradually expanded in the plant lineage during evolution. Although Arabidopsis *Nup98* paralogs were reported to be functionally redundant in regulating flowering time and starch degradation (44, 45), the duplication of *Nup98* may potentially drive differential assembly of the NPC and allow specific regulation of immune-related cargo transport under distinct environmental/stress conditions. Indeed, Arabidopsis *Nup98a* and *Nup98b* display partially different expression patterns under various developmental and pathological contexts (e-Northern Expression Browser in bar.utoronto.ca) (46). However, whether different Nup98 paralogs are collaboratively or differentially incorporated into the NPC awaits future investigation.

In addition to Nup98 and Nup96, a recent study by de Leone et al. reported that perturbing the IRC nucleoporin Nup205 attenuates plant immunity by affecting the expression of a core clock gene family *NIGHT LIGHT-INDUCIBLE AND CLOCK-REGULATED (LNK*) during pathogen infection (47) (**Figure 1**). This finding is consistent with the emerging concept that the circadian rhythm is integrated with plant immune regulatory pathways (48, 49) and underscores a role of the NPC in this process. The nucleoporin’s influence on clock genes was also observed in Drosophila. Two interacting nuclear basket nucleoporins, Tpr (Megator) and Nup153, set the pace of the clock in Drosophila by regulating the protein ubiquitylation/stability and nuclear translocation of the core oscillator PERIOD and TIMELESS (50, 51). Though the roles of Tpr and Nup153 during immune activation in flies are not clear, they appear to be involved in the insertion of HIV-1 DNA into the human genome region that is close to the nuclear pore, a location known to associate with transcriptionally active chromatin (52). Consistently, depletion of Tpr inhibited HIV gene expression in infected cells (53). Additionally, the HIV-1 capsid is physically associated with Nup153, which is required to facilitate the viral import into the nucleus (53, 54).

Although the structure and composition of the NPC are highly conserved among eukaryotes, plants have evolved several specific nucleoporins, which, interestingly, all appear to play a role in plant immune responses. Constitutive Expressor of Pathogenesis-Related Genes 5 (CPR5), a plant unique nucleoporin localized in the NPC membrane module, is involved in gating ETI activation in Arabidopsis. CPR5 switches from oligomer to monomer upon NLR immunoreceptor activation and releases the cyclin-dependent kinase inhibitors SIAMESE and SIAMESE-RELATED 1 to trigger a noncanonical activation of cell cycle transcription factor E2Fs that can drive defense gene expression (55–57) (**Figure 1**). CPR5 was recently found to interact with PLANT NUCLEAR ENVELOPE TRANSMEMBRANE 1 (PNET1), a novel plant nucleoporin that also exists in animals (58). Interestingly, the function of the human PNET1 homolog has also been linked with the cell cycle and was found to promote the proliferation of lung cancer cells (59). Cell cycle regulators are involved in both effector-triggered cell death in plants and apoptosis in mammals (60, 61). Because both CPR5 and PNET1 connect with cell cycle regulatory pathways, the interaction between these two proteins would be interesting for further investigation to reveal the interplay between the cell cycle and immune pathway at the nuclear pore. Another angiosperm-specific nucleoporin, Nup82, interacts with its homolog Nup136 at the nuclear basket. They are redundantly required for SA-dependent immune responses. The *nup82 nup136* double mutant plants are impaired in benzothiadiazole (an analog of SA)-induced resistance to *P. syringae* (62). It is worth noting that the NPC nuclear basket is intimately associated with the nucleoskeleton (63) and that mutations in nucleoskeleton genes also cause altered SA responses in Arabidopsis (64). The influence of nucleoskeleton on plant immune will be further discussed later.

## The Role of Nuclear Transport Receptors and Their Cargo Specificity in Modulating Plant Immunity

The selective transport of macromolecules through the NPC mainly depends on karyopherin proteins, a superfamily of nuclear transport receptors (NTRs), including importins and exportins. Karyopherin-α, also known as importin-α, is an adaptor protein that connects nuclear localization signal (NLS)-containing protein cargo with a karyopherin-β protein (65). Karyopherin-β proteins are capable of directly interacting with FG nucleoporins and mediate the translocation of the importin-α/cargo complex across the NPC (66, 67). Karyopherin-β can also directly interact with some cargo molecules and mediate their nuclear shuttling independent of a karyopherin-α (68–70). Depending on the transport direction, karyopherin-βs are categorized into importins and exportins. The karyopherin-β family was thought to be established before the divergence of eukaryotic life and has been largely maintained since. In contrast, importin-α proteins diverged early in eukaryotes, evolved within lineages, and have expanded dramatically with the complexity of organisms (71). Accumulating evidence suggests that different karyopherins may have evolved to mediate the transport of specific cargo populations and thus participate in regulating distinct cellular processes (65, 69, 72). Multiple karyopherin-β proteins in Arabidopsis have been reported to function in plant immune regulation, and this role seems mostly attributed to their cargo specificity.

Proximity-labeling proteomics identified transcription corepressors TOPLESS (TPL) and TPL-RELATED proteins (TPRs), which are negative regulators of defense activation, as specific cargo of Exportin-4 (XPO4) in Arabidopsis. XPO4 facilitates the nuclear export of TPR1 in the presence of high levels of SA to negatively regulate the SA-mediated immune amplification (**Figure 1**). Consistently, loss of *XPO4* dramatically enhances *cpr5*-dependent autoimmune activation in Arabidopsis (73). Intriguingly, *xpo4* single mutants are compromised in basal resistance and NLR-mediated immune activation, suggesting distinct regulatory roles of this exportin at multiple levels of plant immune induction.

Mutations in another karyopherin-β, *KA120*, leads to a typical NLR-dependent auto-immune response in the absence of pathogens. KA120 is required to restrict the nuclear activity of SNC1 and is essential to prevent SNC1 autoactivation in the nucleus (**Figure 1**). Overexpression of KA120 efficiently suppresses SNC1-dependent autoimmunity and knocking down *SNC1* partially rescues the *ka120*-associated autoimmune phenotype (74). However, whether SNC1 is a direct cargo of KA120 is yet to be determined. The human homolog of KA120, importin-11 (IPO11), has not been reported to be directly involved in immune regulation. However, IPO11 was found to be a tumor suppressor by maintaining the protein level of Phosphatase and Tensin Homologue (PTEN) (75). Loss of *IPO11* leads to degradation of PTEN, and deletion, mutation, and suppression of *PTEN* are often associated with cancers in various tissues (76).

MOS14 is a predicted importin that transports serine/arginine-rich (SR) proteins (77), which are required for the recognition of splice sites and spliceosome assembly. The loss-of-function *mos14* mutation alters the splicing pattern of multiple NLR genes, including *SNC1*, which leads to compromised immunity (78) (**Figure 1**). Aside from importing SR proteins, the human homolog of MOS14, TNPO3, is also responsible for HIV nuclear import. TNPO3 can interact with the HIV1 integrase, which facilitates host integration of HIV-1 DNA, and disrupting this integrase-TNPO3 interaction can block HIV nuclear import (79, 80).

XPO1 is a conserved exportin across the eukaryotes that binds the leucine-rich nuclear export signal (NES) to mediate protein nuclear export. In animals, XPO1 can interact with NF-κB, a protein complex that is important to activate immune response during infection. The nuclear export of NF-κB is an essential step for preventing autoimmune diseases by controlling the level of activated NF-κB in the nucleus (81). In Arabidopsis, XPO1 is required for maintaining the nucleocytoplasmic balance of EDS1, which is important for ETI activation (82). Recently, Zhang etal. (83) reported that XPO1 is a susceptibility factor in Arabidopsis and *Nicotiana benthamiana* for resistance against the turnip mosaic virus (TuMV). XPO1 promotes TuMV infection by mediating the nuclear export of the viral replicase to facilitate its accumulation in the viral replication complexes outside of the nucleus. XPO1 also exports other sumoylated host factors to attenuate host immune responses (83).

Besides karyopherin-βs, certain importin-αs also show a specific role in immune regulation. For example, importin-α3/MOS6 has been implicated as a specific import adapter for SNC1 (84) (**Figure 1**). Loss of *MOS6*, but not any of the other eight importin-αs encoded by Arabidopsis, partially suppresses the autoimmune phenotype induced by SNC1. Moreover, SNC1 preferably interacts with MOS6 among all importin-αs in coimmunoprecipitation assays performed using a heterologous transient expression system (84). However, SNC1 harbors only weakly predicted NLS sequences, and whether these sequences are required for SNC1-MOS6 interaction is not clear. MOS6 also interacts with another NLR protein TIR-NB13 (TN13), implying a specialized function of MOS6 in mediating the nuclear transport of NLR proteins (85).

In animals, targeting NTRs has been used therapeutically to alleviate symptoms or enhance immunity. For example, importin-α3 is responsible for transporting the transcription factor NF-κB into the nucleus to coordinate expression of inflammatory genes, and vitamin D supplementation can reduce the airway hyperresponsiveness and allergic airway inflammation in ovalbumin-sensitized and -challenged mice by downregulating the protein level of importin-α3 (86, 87). Inhibiting human XPO1 can enhance innate immunity by increasing nuclear accumulation of p62, which promotes expression of innate immune-related genes. This eventually leads to reduced Kaposi’s sarcoma-associated herpesvirus (KSHV) lytic replication (88). Therefore, NTRs play a vital role in selectively maintaining the protein homeostasis and activity of key immune regulatory proteins in both plants and animals. We envision that targeted chemical or genetic manipulation of functionally specialized plant NTRs may enable us to specifically modulate nuclear transport kinetics and signaling strength to achieve desired plant immune outcomes without affecting the bulk nuclear transport.

## The Nuclear Lamina Is an Emerging Player in Nuclear Membrane-Associated Immune Regulation

Lamin and lamin-like proteins together with other proteins associated with the nucleoskeleton form a dense fibrous protein meshwork that is located beneath the inner nuclear membrane (INM) and called the nuclear lamina (NL). NL components not only regulate nuclear structure and mechanics but also play essential roles in a variety of cellular processes, including cellular signaling, gene transcription, epigenetic regulation, cell cycle progression, and cell differentiation (89–91). Lamins and nuclear matrix constituent proteins (NMCPs) are the major constituent of the nucleoskeleton in animals and plants respectively. Lamins and NMCPs share a similar domain architecture with a central coiled-coil rod domain flanked by a short N-terminal head and a long C-terminal tail domain that contains an NLS sequence (92). However, lamins and NMCPs are likely to have evolved independently as part of the nucleoskeleton, as the origin of NMCPs occurred with the divergence of Charophyta (93). In Arabidopsis, NMCPs are also known as CROWED NUCLEI (CRWN) proteins, including CRWN1, CRWN2, CRWN3, and CRWN4 (94, 95). Both lamins and CRWNs have been reported to play a role in regulating immune responses.

In Arabidopsis, mutations in *CRWN1* result in accumulation of JA, up-regulated JA-responsive gene expression, and enhanced resistance against necrotrophic pathogen *B. cinerea*. Interestingly, *crwn1* mutants also display compromised PTI but intact basal resistance against the hemibiotrophic bacterial pathogen *P. syringae*, suggesting a complicated role of CRWN1 in regulating plant responses against different types of pathogens (96). Adding more complexity, higher-order *crwn* mutants, including *crwn1 crwn2* and *crwn1 crwn4*, showed significantly enhanced SA-mediated transcriptional response, enhanced resistance against *P. syringae*, as well as activation of other abiotic stress responses (64). Consistently, a recent study showed that loss of *NMCP* genes in liverwort *Marchantia polymorpha* activates both biotic and abiotic transcriptome responses (97). These studies suggest that the nucleoskeleton integrity is critical for maintaining the homeostasis of plant stress responses. In the Arabidopsis *crwn1 crwn2* double mutant, the levels of histone H3 lysine 27 trimethylation (H3K27me3), which is often linked to transcriptional repression, are reduced near *SAR DEFICIENT 1* (*SARD1*) and *CALMODULIN BINDING PROTEIN 60-LIKE.G* (*CBP60g*), two genes that encode critical transcription factors to promote SA biosynthesis, providing a potential mechanism for the enhanced SA responses in *crwn* mutants (98). Consistently, it was demonstrated that CRWN1 is physically associated with Polycomb Repressive Complex 2 (PRC2), which mediates H3K27me3 modification (99) (**Figure 1**). In addition, the carboxyl terminus of CRWN1 interacts with and enhances the DNA-binding activity of NAC WITH TRANSMEMBRANE MOTIF1-LIKE9 (NTL9), a transcription factor involved in repressing the expression of the *PATHOGENESIS-RELATED1* (*PR1*) gene (100) (**Figure 1**). These findings reveal a critical and previously underappreciated role for the nucleoskeleton in regulating plant immunity at the epigenetic and transcriptional level.

In animals, lamin A/C plays a prominent role in multiple functional aspects of immune cells, including cell development and differentiation, nuclear permeability, and cell migration (101). For example, neutrophils can reduce the nuclear stiffness by almost completely losing their lamin A/C during differentiation (102, 103). This generates lobed nuclei in neutrophils, which facilitate neutrophils passing through narrow spaces during migration (104). On the other hand, overaccumulation of lamin A/C in adipose tissue macrophages promotes NF-κB nuclear translocation and increases the expression of proinflammatory genes that lead to the activation of innate immune response (105). Intriguingly, *P. syringae* infection led to a decline of the CRWN1 protein level within hours, and SA treatment also triggered rapid proteosome-dependent degradation of CRWN1 in Arabidopsis, potentially facilitating defense activation (100). These studies suggest that modulating nucleoskeleton dynamics may be a convergent strategy to orchestrate immune activities in eukaryotes.

## Conclusions and Future Perspective

Although not located at the frontline of host-microbe interactions, the nucleus is one of the most critical downstream convergent points for immune signaling. As the boundary of the nucleus, NE-associated core molecular machinery and mechanisms, including structurally and functionally conserved nucleoporins, nuclear transport receptors, and nucleoskeletal proteins, are intimately involved in coordinating immune activation and amplitude in plants (Summarized in **Figure 1**). Future investigations will further advance our understanding of critical questions that remain in the field, such as how nucleoporins may regulate different immune pathways and defense responses by differentially affecting the NPC function, what is the immune-related substrate spectrum of NTRs and how it is determined and regulated at the molecular level, and how other components of the nuclear lamina may influence chromatin activity at the biochemical and epigenetic level to determine defense gene expression. Moreover, we currently have very little information about how integral plant NE proteins, a population of transmembrane proteins with a variety of prominent functions and activities at the NE (e.g. calcium transport, mechanosensation, and chromatin organization), may participate in plant immune regulation. Recently, PNET2, a conserved INM transmembrane protein in Arabidopsis, was shown to interact with CRWN1 and play a role in regulating higher order chromatin architecture at the nuclear periphery and coordinating the activation of plant stress responses including immunity (106). A more comprehensive understanding of the plant NE protein composition and subsequent detailed functional investigation of NE proteins will provide new insight into the NE-centered immune regulatory mechanism in plants.

## Author Contributions

YF and YG wrote the article. All authors contributed to the article and approved the submitted version.

## Funding

This work was supported by the Arnon Graduate Fellowship (to YF) and the USDA National Institute of Food and Agriculture (HATCH project CA-B-PLB-0243-H), the National Science Foundation (MCB-2049931), Hellman Fellows Fund, and startup funds from Innovative Genomics Institute and University of California Berkeley (to YG).

## Conflict of Interest

The authors declare that the research was conducted in the absence of any commercial or financial relationships that could be construed as a potential conflict of interest.

## Publisher’s Note

All claims expressed in this article are solely those of the authors and do not necessarily represent those of their affiliated organizations, or those of the publisher, the editors and the reviewers. Any product that may be evaluated in this article, or claim that may be made by its manufacturer, is not guaranteed or endorsed by the publisher.
